# Diffusion tensor free water MRI predicts progression of FLAIR white matter hyperintensities after ischemic stroke

**DOI:** 10.3389/fneur.2023.1172031

**Published:** 2023-09-22

**Authors:** Kyle C. Kern, Marwah S. Zagzoug, Rebecca F. Gottesman, Clinton B. Wright, Richard Leigh

**Affiliations:** ^1^Stroke Branch, National Institute of Neurological Disorders and Stroke, National Institutes of Health, Bethesda, MD, United States; ^2^Department of Neurology, Johns Hopkins Medicine, Baltimore, MD, United States

**Keywords:** white matter hyperintensities, cerebral small vessel disease, ischemic stroke, diffusion tensor imaging, free water

## Abstract

**Background:**

The progression of FLAIR white matter hyperintensities (WMHs) on MRI heralds vascular-mediated cognitive decline. Even before FLAIR WMH progression, adjacent normal appearing white matter (NAWM) already demonstrates microstructural deterioration on diffusion tensor imaging (DTI). We hypothesized that elevated DTI free water (FW) would precede FLAIR WMH progression, implicating interstitial fluid accumulation as a key pathological step in the progression of cerebral small vessel disease.

**Methods:**

Participants at least 3 months after an ischemic stroke or TIA with WMH on MRI underwent serial brain MRIs every 3 months over the subsequent year. For each participant, the WMHs were automatically segmented, serial MRIs were aligned, and a region of WMH penumbra tissue at risk was defined by dilating lesions at any time point and subtracting baseline lesions. Penumbra voxels were classified as either stable or progressing to WMH if they were segmented as new lesions and demonstrated increasing FLAIR intensity over time. Aligned DTI images included FW and FW-corrected fractional anisotropy (FA_Tissue_) and mean diffusivity (MD_Tissue_). Logistic regression and area under the receiver-operator characteristic curve (AUC) were used to test whether baseline DTI predicted voxel-wise classification of stable penumbra or progression to WMH while covarying for clinical risk factors.

**Results:**

In the included participants (*n* = 26, mean age 71 ± 9 years, 31% female), we detected a median annual voxel-wise WMH growth of 2.9 ± 2.6 ml. Each baseline DTI metric was associated with lesion progression in the penumbra, but FW had the greatest AUC of 0.732 (0.730 – 0.733) for predicting voxel-wise WMH progression pooled across participants.

**Discussion:**

Baseline increased interstitial fluid, estimated as FW on DTI, predicted the progression of NAWM to WMH over the following year. These results implicate the presence of FW in the pathogenesis of cerebral small vessel disease progression.

## Introduction

White matter hyperintensity (WMH) lesions detected on FLAIR MRI are a hallmark of chronic cerebrovascular disease and vascular contributions to cognitive impairment and dementia (VCID). WMHs are associated with risk for recurrent stroke, (1) cognitive decline, (2) and incident dementia (3). Progression of WMH is also associated with cognitive decline (2) and conversion to mild cognitive impairment or dementia (4). Progressive WMH accumulation implicates cerebral small vessel disease (SVD), although WMHs are also seen in other pathologies including Alzheimer's disease. The pathophysiology of WMH progression is unclear but may be due to arteriolosclerosis, subclinical ischemia, endothelial damage with blood-brain barrier (BBB) disruption, (5) vasogenic edema, (6) venous stasis or collagenosis, (7) demyelination, and axonal injury (8). Although there are no known therapies that directly affect the progression of WMH, a better understanding of the pathophysiological changes detected on imaging preceding progression could facilitate future therapeutic development.

In patients with stroke and chronic cerebrovascular disease, imaging has helped elucidate the changes leading to WMH progression. The normal appearing white matter (NAWM) immediately surrounding FLAIR WMH is termed the WMH “penumbra” and is at greatest risk for progression into overt FLAIR WMH lesions (9). Diffusion tensor imaging (DTI) can detect impaired white matter integrity even in the NAWM, characterized by reduced fractional anisotropy (FA) and increased mean diffusivity (MD) (9). DTI measures the directional diffusion of water in tissue and is more sensitive to demyelination, axonal damage, or fluid shifts than conventional MRI. In the WMH penumbra, increased MD and reduced FA can be detected, extending far past the edge of visible WMH on FLAIR (9–14). In a longitudinal cohort study of elderly volunteers over 18 months, new WMH progression occurred mostly within 3 mm of the edge of periventricular WMH, with no new WMH voxels detected past 5 mm (15). Other longitudinal studies have found that alterations in MD and FA in the WMH penumbra predict the progression of NAWM into the new WMH (10, 16, 17).

More complex DTI models can further elucidate white matter microstructure by modeling distinct tissue compartments. DTI free water (FW) imaging models the diffusion signal as having two components: a fast, freely moving (isotropic) diffusion signal that reflects a CSF-like compartment and a slower, hindered diffusion signal that reflects tissue architecture (18). Within white matter, the isolated CSF-like signal is estimated as the FW fraction. The other component of the diffusion signal modeled is the tissue tensor compartment, which can provide FW-corrected measures of WM integrity: FA_Tissue_ and MD_Tissue_. These FW-corrected measures of white matter integrity may more closely reflect the integrity of underlying white matter after removing the influence of free water and CSF partial volume effects. The estimated FW signal within white matter is also relevant to the underlying physiology since it is thought to reflect interstitial fluid. Increased DTI FW in white matter is closely associated with chronic cerebrovascular disease and VCID. FW measurements outperformed conventional imaging markers of cerebral SVD in predicting disability and processing speed in sporadic SVD and CADASIL patients (19). FW is also altered in the NAWM of the WMH penumbra, suggesting altered FW is an early precursor to WMH progression (20).

In this study of participants with prior ischemic stroke or TIA and evidence of early confluent WMH on MRI, we measured longitudinal changes in white matter FW and tested the hypothesis that increased FW is a predictor of NAWM progression to WMH remote from the infarct.

## Methods

### Human research protocol

This study was approved by the Ethics and Institutional Review Boards of NINDS/NIH (IRB Protocol Number: 18N0020), Suburban Hospital, Johns Hopkins Medicine, Bethesda, MD, and MedStar Washington Hospital Center, Washington, DC (Protocol Number: 2018-093) and is registered with clinicaltrials.gov (NCT03366129). All subjects provided written informed consent.

### Data integrity and availability

The corresponding author oversaw the integrity of data collection and analysis. Data will be made available upon reasonable request to the corresponding author after close-out and publication of the parent study under a formal data-sharing agreement and with approval from the requesting researcher's local ethics committee. The software used is freely available for non-commercial use (Freesurfer, FSL, DIPY, QIT) or commercially available (STATA).

### Study population

Patients with a history of ischemic stroke or TIA were eligible for enrollment in this observational study if a prior MRI showed evidence of chronic SVD, defined on FLAIR MRI as early confluence of WMH in regions remote from any visible infarct. Participants were excluded if they were unable to tolerate MRI, could not ambulate without assistance, could not communicate, had severe dementia as determined by a cognitive screening test, or had concurrent neurologic disease predisposing to WMH of non-cerebrovascular origin. Characteristics of the index ischemic event were derived from the medical chart and a review of available images. The etiology of the index ischemic event was classified using the TOAST classification (21). The first research MRI occurred at least 3 months after a prior stroke or TIA, and serial scans were acquired every 3 months for the following year. Due to disruptions in research surrounding the COVID-19 pandemic, some time points were missed. For participants that missed their 1-year time point, MRIs performed during the second year of the study were used to quantify WMH lesion growth. The data were acquired between September 2018 and October 2022.

### MRI protocol

For each participant, brain MRIs were acquired serially on the same scanner at one of two hospital sites using a 3T Siemens Skyra or a 3T Phillips Achieva. The acquisition included a 3D T1 (resolution 1 × 1 × 1 mm), a 3D FLAIR (resolution 1 × 1 × 1 mm), an axial FLAIR (resolution ~1 × 1 × 3.5 mm), DTI (Siemens: 12 directional images with B = 1,000 s/mm^2^, 4 B = 0 images, resolution 1.7 × 1.7 × 3.5 mm or Phillips: 15 directions at B = 1,000 s/mm^2^, 1 B = 0 image, 2 × 2 × 3.5 mm), and a susceptibility-weighted image (SWI) (Siemens: 0.86 × 0.86 × 1.5 mm or Phillips: 0.57 × 0.57 × 2 mm) (see Supplementary Methods).

### MRI processing

MRI data were analyzed using FreeSurfer v7.2 (freesurfer.net), (22) the FMRIB Software Library v6.0 (fsl.fmrib.ox.ac.uk), (23) DIPY 1.4 (dipy.org), (24) and the Quantitative Imaging Toolkit (cabeen.io/qitwiki) (25).

### DTI processing

After correction for motion and eddy current distortions using FSL (26), a single-shell FW model was fit using DIPY (27, 28). The resulting images included the FW fraction, fractional anisotropy corrected for FW (FA_Tissue_), mean diffusivity corrected for FW (MD_Tissue_), and a trace image calculated as the geometric mean of the diffusion-weighted images.

### Alignment of longitudinal images

For each time point, 3D FLAIR images were rigidly aligned to the T1. DTI was aligned within-subject to the T1 first using rigid boundary-based registration with the trace (29) and then non-linearly using the FA. This approach provided the best anatomical alignment in the periventricular regions, where most WMH occurs. For each participant, all time point T1s were rigidly aligned to a midpoint space equidistant from all time points (to minimize longitudinal alignment bias) and averaged. For each modality, transforms to T1 and then to midpoint were combined, interpolating only once.

### Infarcts, lesions, and tissue segmentation

Chronic infarcts were manually segmented on baseline 3D FLAIR and T1 with reference to acute MRI when available to exclude them from the regions of interest (ROI) used for analysis. The FLAIR hyperintense regions surrounding infarcts were also excluded from white matter and lesion masks. T1, 3D FLAIR, and axial FLAIR were used to segment WMH, white matter, gray matter, and CSF for each time point using FreeSurfer-based SAMSEG (Supplementary Methods) (22). Segmentations were visually inspected and manually corrected for accuracy and to exclude FLAIR hyperintensity associated with chronic infarcts. Lesions at any time point were combined across time, the summation mask was dilated by 3 mm, and then the baseline WMH was subtracted to create a WMH penumbra for both baseline and de novo lesions remote from chronic infarcts. The WMH penumbra is at greatest risk for WMH progression in SVD, so it provides an ideal region to study the imaging changes that precede new WMH development. A prior longitudinal analysis found that most new WMH progression occurred within 3 mm of the periventricular WMH edge, and none occurred past 5 mm over a median of 18 months (15). The remaining non-lesion, non-penumbra white matter comprised the NAWM mask that remained stable throughout the study period. Cerebral microbleeds (CMBs) and lacunes were counted on SWI and FLAIR, respectively, according to established criteria (30, 31). Chronic cortical infarcts were also identified on baseline images using FLAIR and T1 as wedge-shaped FLAIR hyperintense lesions with T1 cortical hypointensity at least 5 mm in diameter in a vascular territory.

### Localizing lesion progression

**Figure 1:**
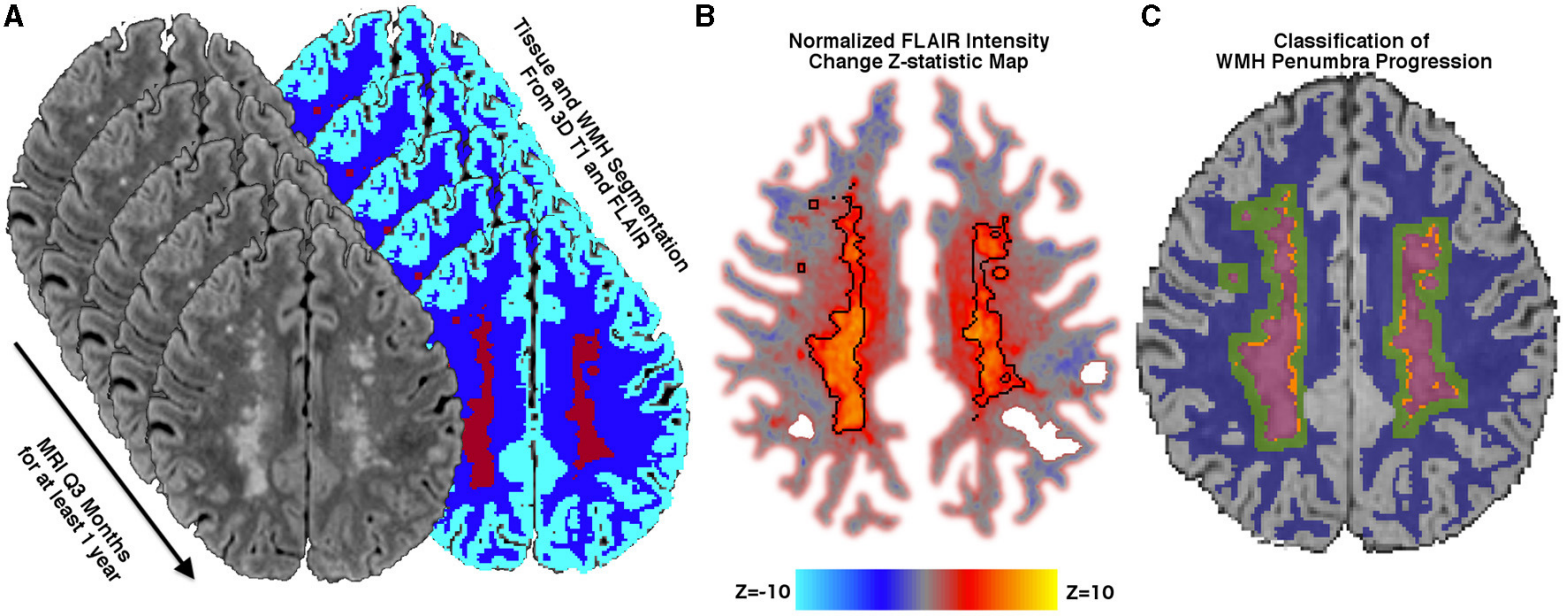
Methods. **(A)** Tissue segmentation using FLAIR and T1 into the cerebrospinal fluid, normal appearing white matter (NAWM) (blue), gray matter (cyan), and white matter hyperintensities (WMH) (maroon). 3D FLAIR is normalized to mean gray matter signal intensity and then rigidly aligned to a midpoint space. **(B)** Normalized FLAIR intensity is regressed over time, and the slope is converted into a Z-statistic map using the mean and standard deviation of the slope within stable NAWM tissue. A Z-statistic map is shown with baseline lesions outlined in black. The Z-statistic map is thresholded and cluster-corrected with Z ≥ 2.3 and *p* < 0.01. **(C)** WMH lesions at any time point are dilated by 3 mm, and baseline WMHs (maroon) are subtracted, thereby creating a baseline WMH penumbra. Penumbra WM voxels that are segmented as new WMHs at any subsequent time point *and* demonstrate significantly increasing normalized FLAIR intensity over time are classified as new WMH lesion growth (orange), while voxels not meeting these criteria are classified as WMH penumbra: No growth (green).

FLAIR lesion progression was determined empirically by calculating voxel-wise FLAIR intensity changes over time rather than relying on image segmentation alone. To accomplish this and permit longitudinal comparisons across scan sessions, at each time point, FLAIR intensity was normalized to the mean signal intensity of the gray matter and then denoised using a non-local means filter (25). Within the WMH penumbra, the slope of FLAIR intensity change over time was calculated at each white matter voxel using linear regression. Similar to the lesion difference method employed by Schmidt et al. (32) the regression coefficient at each voxel was converted to a Z-statistic (Figure 1) using the mean and standard deviation of the regression coefficients within stable NAWM. Z-statistics were cluster-corrected using thresholds *Z* ≥ 2.3 and *p* < 0.01 based on Gaussian Random Field Theory. Regions of significant FLAIR signal change were reviewed for both increasing and decreasing intensity changes. At the border of segmented WMH and the surrounding penumbra, there were no regions that significantly decreased in FLAIR signal intensity over the study period, so WMH penumbra voxels were classified as WMH progression or stable. Penumbra voxels demonstrating increasing FLAIR signal intensity and segmented as new lesions at later time points were classified as WMH lesion progression, while all remaining penumbra voxels were classified as stable.

### Statistical analysis

Statistical analysis was performed using STATA v17.0 (stata.com). Mean and standard deviation were used for normally distributed variables, while median and interquartile range were used for non-normally distributed variables. Multiple linear regression was used to identify clinical predictors of WMH growth, considering baseline WMH, age, sex, and race. Simple regression was used to test the relationship between WMH growth and additional imaging markers such as cerebral microbleeds, lacunes, and chronic infarct volume.

#### Region of interest approach

Across four white matter ROIs (NAWM, penumbra: no growth, WMH progression, and baseline lesions), baseline differences in normalized FLAIR intensity and DTI metrics (FW, FA_Tissue_, and MD_Tissue_) were assessed with repeated-measures ANOVA and a *post-hoc* Tukey's test. Separate mixed-effects linear regression models were used to estimate the mean change over time for each DTI metric or FLAIR signal intensity within these tissue ROIs. Fixed effects included tissue ROI (NAWM as reference), time, hospital site (due to potential scanner differences), age, sex, race (Black, Hispanic, or other), and baseline WMH volume, while random effects included intercepts for participants. Change within ROIs was identified as a tissue-by-time interaction, and pairwise comparisons were performed with Bonferroni correction. Residuals were inspected for normality and heteroscedasticity.

#### Voxel-wise approach

Within the WMH penumbra, each voxel was classified as either new WMH lesion progression or no growth over the study period using the voxel-wise analysis described above. For each DTI metric, logistic regression was used to test whether baseline metrics predicted voxel-wise classification of progression to WMH across all participants' pooled penumbra voxels while covarying for age, sex, race, and baseline WMH volume. Clustering of standard errors was incorporated to adjust for the non-independence of voxels within the same participant.

Receiver-operator characteristic (ROC) curve analysis was used to quantify the classification performance of each baseline DTI metric's logistic regression to predict voxel-wise WMH progression. The resulting area under the ROC curve (AUC) was used to compare the classification performance of the baseline DTI metrics, and bootstrap resampling was used to calculate 95% confidence intervals. We also report log-likelihood R^2^ values (pseudo R^2^) from the logistic regression analyses.

## Results

### Participants

A total of 26 participants with at least 1-year follow-up were included in this analysis (see Table 1) after excluding 1 participant with an interval ischemic stroke. Participants had a median of 4 visits (IQR 2) with a median visit-to-visit interval of 126 (IQR 95) days. The index ischemic event was stroke in 24 participants and TIA in 2 participants. For two participants with TIA, one had a chronic subcortical infarct (i.e., lacune) at presentation, and the other had a transient territorial perfusion delay that localized with the presenting symptoms. The etiology of index ischemic events was classified as small-vessel occlusion (SVO) in 9 (35%), cardioembolism (CE) in 6 (23%), large-artery atherosclerosis (LAA) in 4 (15%), and undetermined etiology in 7 (27%) participants. The median time from the index ischemic event to baseline imaging was 116 (IQR 61) days. On baseline imaging, CMBs were present in 7 (27%), lacunes were present in 10 (38%), and chronic cortical infarcts were present in 8 (31%) participants.

**Table 1:** Participant characteristics.

***N***	**26**
Mean age (SD)	71 (9)
Male:Female	18:8
**Race/Ethnicity**	
White	21 (81%)
White-Hispanic	2 (8%)
Black	4 (15%)
Asian	1 (4%)
**Mean # CV Risk Factors (IQR)**	2 (1)
Hyperlipidemia	20 (76%)
Hypertension	19 (73%)
Atrial fibrillation	8 (31%)
Diabetes	6 (23%)
Obstructive Sleep Apnea	5 (19%)
Tobacco use (in past 5 years)	4 (15%)
Coronary artery disease	2 (8%)
Prior stroke (%)	25 (96%)
Two or more strokes (%)	7 (27%)
Prior TIA (%)	5 (19%)
Median months since stroke/TIA (IQR)	4 (2)
Median months study duration (IQR)	18 (6)
Median study visits (IQR)	4 (2)
Median baseline NIH stroke scale (IQR)	0 (1)
Median baseline modified rankin score	1 (1)
Median chronic infarct volume ml (IQR)	0.7 (1.8)
Median baseline WMH volume ml/% of TICV (IQR)	16 (19)/1.0% (1.2)
Median voxel-wise WMH growth ml/% of TICV (IQR)	2.9 (2.6)/0.22% (0.19)

SD, standard deviation; IQR, interquartile range; CV, cerebrovascular; TIA, transient ischemic attack; WMH, white matter hyperintensity; TICV, total intracranial volume.

### White matter hyperintensity progression

Using voxel-wise analysis of FLAIR signal intensity change to confirm new WMH, mean WMH growth was 2.9 ± 2.6 ml per year (0.22 ± 0.19% of TICV). There were no regions where FLAIR signal intensity significantly decreased over the course of the study. Over 1 year, WMH progression involved a median of 8% of the 3 mm WMH penumbra volume (IQR 6%, range 3–21%). Annualized WMH growth was associated with baseline WMH volume [beta 0.123 (0.050–0.196), *p* = 0.002] but not age, sex, or race. There were no group differences in annualized WMH growth for those with and without CMBs, with and without lacunes, nor with and without cortical infarcts. There were no group differences in WMH growth by the etiology of the index ischemic events (SVO, CE, or LAA).

### DTI changes in white matter regions of interest

**Figure 2:**
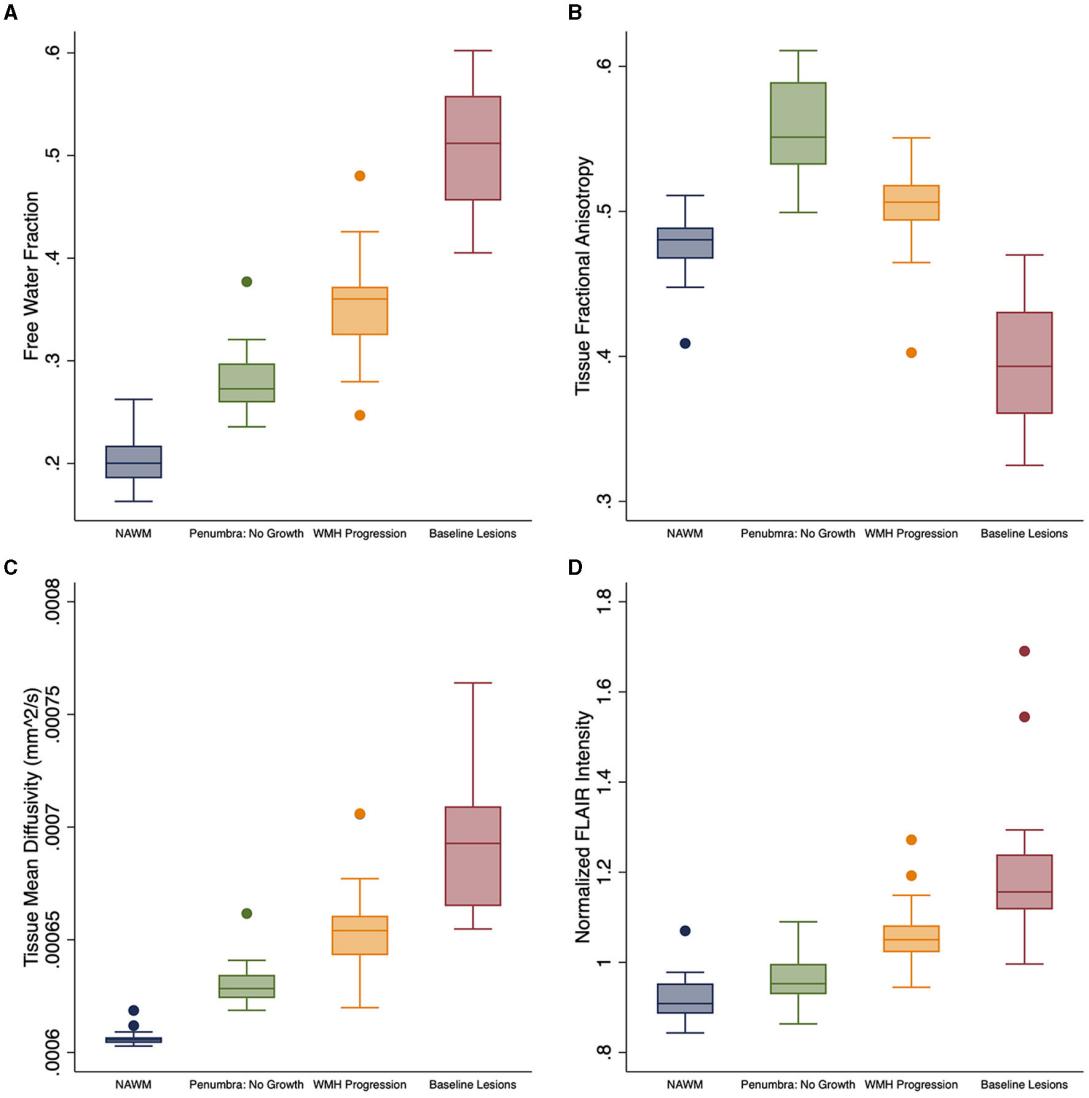
Baseline white matter DTI differences. Baseline differences across tissue regions for white matter measures: **(A)** free water (FW), **(B)** FA_Tissue_, **(C)** MD_Tissue_, and **(D)** normalized FLAIR intensity. FA_Tissue_, FW-corrected fractional anisotropy; MD_Tissue_, FW-corrected mean diffusivity.

**Figure 3:**
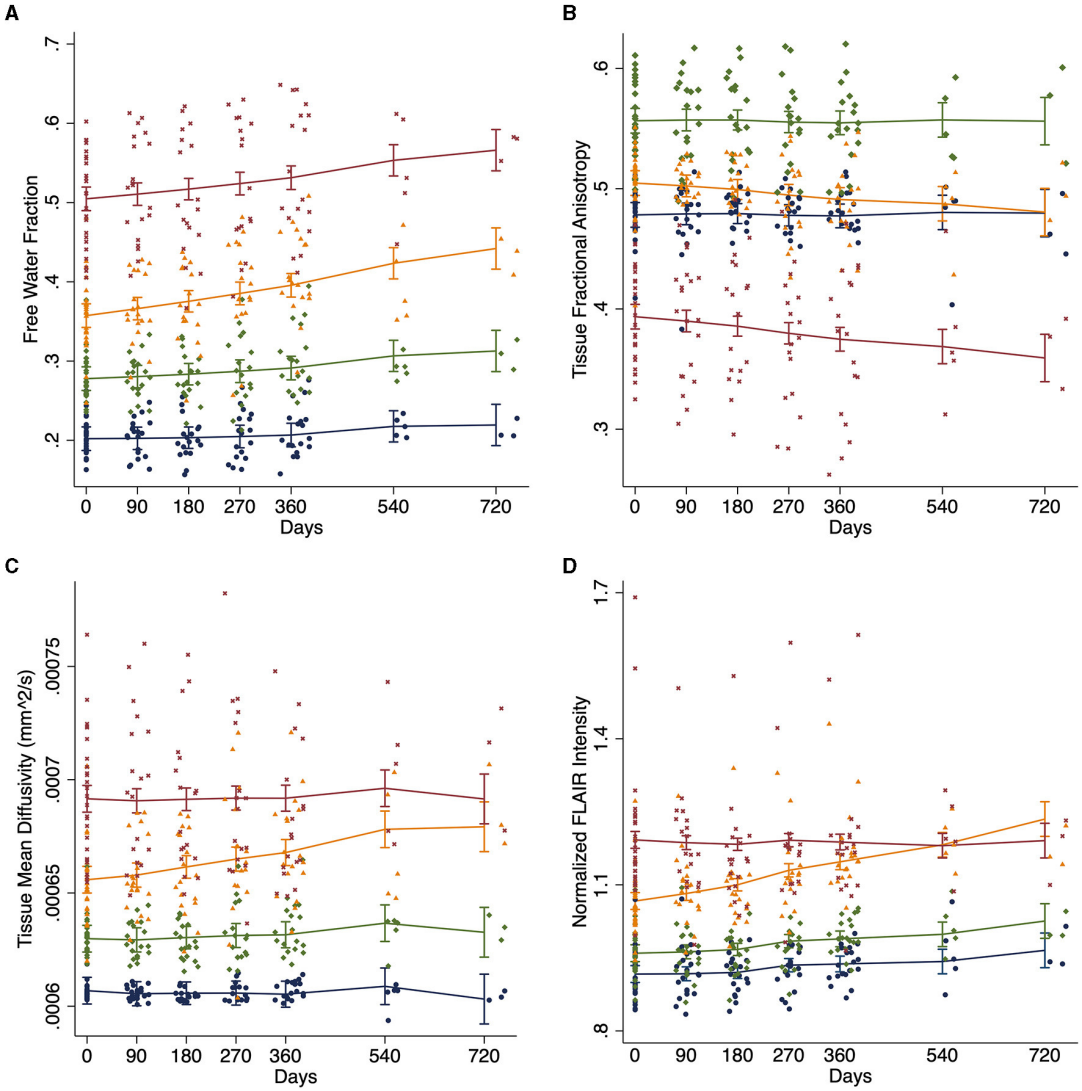
White matter longitudinal changes. Change in white matter measures over time across tissue regions estimated using linear mixed effects models for **(A)** DTI free water, **(B)** tissue fractional anisotropy (FA_Tissue_), **(C)** tissue mean diffusivity (MD_Tissue_), and **(D)** normalized FLAIR intensity. Blue: normal appearing white matter (NAWM); Green: stable WMH penumbra voxels; Orange: WMH penumbra voxels progressing to a new WMH lesion; and Maroon: baseline white matter hyperintensities (WMH).

Free water differed at baseline for each of the four regions [*F*_(3, 25)_ = 534.1; *p* < 0.0001; Table 2 and Figure 2A]. FW increased over time within the penumbra region where new lesion growth was detected and in the baseline WMH lesions (Table 2, Figure 3A). However, after multiple comparison correction, only FW within the new WMH region increased relative to NAWM [0.034/year (0.011–0.057); *p* = 0.001] or the penumbra: no growth region [0.025/year (0.002–0.048); *p* = 0.025]. FA_Tissue_ (corrected for FW) differed at baseline between all regions [*F*_(3, 25)_ = 149.9; *p* < 0.0001; Table 2 and Figure 2B]. FA_Tissue_ decreased over time in the baseline lesions, but this decrease was not significantly more than in other regions (Figure 3B). MD_Tissue_ (corrected for FW) differed between all regions [*F*_(3, 25)_ = 167.5; *p* < 0.0001; Table 2 and Figure 2C]. MD_Tissue_ increased in only the new lesion growth region (Table 2, Figure 3C), and the slope was different from both NAWM (difference: 1.4 × 10^−5^/year [0.23–2.5 × 10^−5^]; *p* = 0.009) and baseline lesions [difference: 1.19 × 10^−5^/year (0.05–2.3 × 10^−5^); *p* = 0.035].

**Table 2:** Baseline and longitudinal change in white matter measures.

**Baseline mean [CI]**	**Baseline WMH**	**New WMH growth**	**Penumbra: no growth**	**NAWM**
FW^a^	0.520 [0.506–0.533]	0.379 [0.369–0.389]	0.285 [0.279–0.292]	0.205 [0.200–0.210]
${\text{FA}}_{\text{Tissue}}^{\text{b}}$	0.385 [0.375–0.395]	0.499 [0.494–0.504]	0.556 [0.550–0.563]	0.479 [0.475–0.483]
MD_Tissue_ (mm^2^/s)^a^	6.91 × 10^−4^ [6.85–6.97]	6.62 × 10^−4^ [6.58–6.66]	6.30 × 10^−4^ [6.28–6.32]	6.06 × 10^−4^ [6.05–6.06]
Normalized FLAIR^c^	1.189 [1.165–1.213]	1.112 [1.096–1.127]	0.977 [0.969–0.985]	0.928 [0.920–0.937]
**Mean annual change**^d^ **[CI]**	**Baseline WMH vs. NAWM**	**New WMH growth vs. NAWM**	**Penumbra: no growth vs. NAWM**	
FW	0.022^*^ [0.005 to 0.040]	0.034^***^ [0.017 to 0.051]	0.009 [−0.008 to 0.026]	
FA_Tissue_	−0.018^*^ [−0.034 to −0.002]	−0.013 [−0.029 to 0.003]	−0.0009 [−0.017 to 0.015]	
MD_Tissue_ (mm^2^/s)	0.14 × 10^−5^ [−0.7 to 1.0 × 10^−5^]	1.1 × 10^−5**^ [0.5 to 2.1 × 10^−5^]	0.32 × 10^−5^ [−0.50 to 1.1 × 10^−5^]	
Normalized FLAIR	−0.024 [−0.056 to 0.009]	0.058^**^ [0.026 to 0.090]	0.008 [−0.024 to 0.040]	

^a^All groups differ with p <0.001, rmANOVA. ^b^All groups differ with p <0.01, rmANOVA. ^c^All groups differ with p <0.001 except penumbra: No Growth vs. NAWM, rmANOVA. ^d^Mixed effects linear regression was used to estimate mean annual change as a tissue × time interaction, with NAWM as the reference tissue group. ^*^p <0.05. ^**^p <0.01. ^***^p <0.001. CI, 95% confidence interval; WMH, white matter hyperintensity; NAWM, normal appearing white matter; FW, free water; FA_Tissue_, fractional anisotropy corrected for FW; MD_Tissue_, mean diffusivity corrected for FW; FLAIR, fluid attenuated inversion recovery MRI; rmANOVA, repeated-measures ANOVA.

### Normalized FLAIR signal intensity in white matter regions

At baseline, mean normalized FLAIR signal intensity was different within each of the white matter regions [*F*_(3, 25)_ = 92.6, *p* < 0.05, Table 2 and Figure 2D]. As expected, mixed effects linear regression demonstrated an interaction between region and time, such that mean normalized FLAIR intensity increased by 0.058 per year (0.026–0.09) in the WMH penumbra: new lesion growth region (Table 2, Figure 3D). This change was different from NAWM [difference: 0.058 (0.015–0.101); *p* = 0.003], penumbra: no growth [difference: 0.050 (0.006–0.093); *p* = 0.015], and baseline WMH lesions [difference: 0.082 (0.038–0.12); *p* < 0.001].

### Voxel-wise predictors of WMH progression

**Figure 4:**
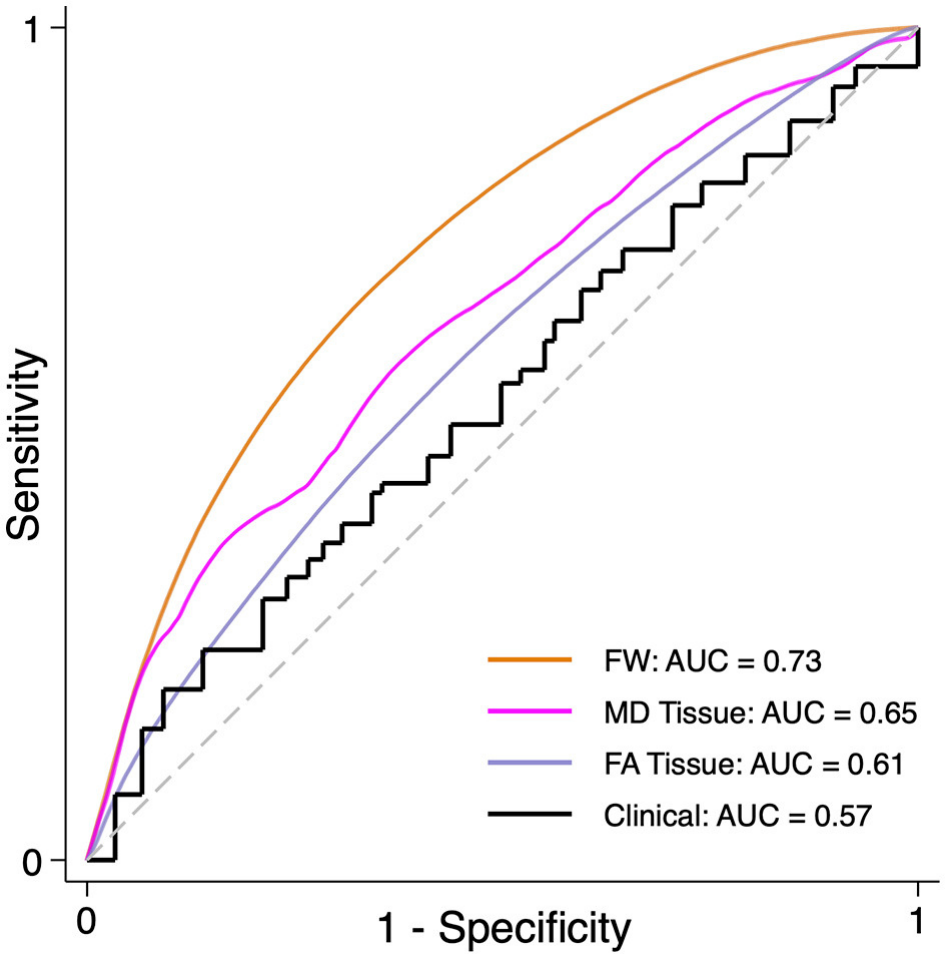
Prediction of WMH progression. Receiver operator characteristic (ROC) curve to classify WMH penumbra voxels, pooled across all participants (*n* = 953,568 voxels) and corrected for within-subject covariance, as stable or progressing to new WMH using clinical predictors in combination with DTI predictors for free water (FW: orange), tissue mean diffusivity (pink), tissue fractional anisotropy (lavender), or clinical predictors alone: age, sex, race, and baseline WMH volume (black). WMH, white matter hyperintensity. AUC, area under the ROC curve.

Using logistic regression across all participants' pooled WMH penumbra voxels and covarying for age, sex, race, MRI site, and baseline WMH lesion volume, each DTI metric at baseline was associated with voxel-wise progression to a new WMH lesion. Baseline voxel-wise FW provided the highest classification performance among DTI metrics with an AUC of 0.732 (0.730–0.733; pseudo R^2^ 0.068; *p* < 0.001) from the ROC curve analysis. Balancing sensitivity and specificity using the Liu method (33), an optimal cutoff point of FW = 0.293 predicted progression to FLAIR WMH lesion over the study period with a sensitivity of 0.70 and a specificity of 0.66. MD_Tissue_ [AUC = 0.633 (0.631–0.635); pseudo R^2^ = 0.020; *p* < 0.001] and FA_Tissue_ [AUC = 0.592 (0.590–0.594); pseudo R^2^ = 0.015; *p* < 0.001] provided less discriminatory power, although they were still associated with voxel-wise WMH lesion progression (Figure 4). Finally, clinical predictors alone (age, sex, race, and baseline WMH volume) did not predict voxel-wise WMH progression[(AUC = 0.543 (0.541–0.545); R^2^ = 0.006; *p* = 0.7].

## Discussion

In this longitudinal study of participants with prior ischemic stroke or TIA, we detected an increasing WMH volume remote from the infarct of 2.9 ml per year over 1–2 years that was preceded by locally elevated DTI FW. Based on our literature review, this is the first study to demonstrate the predictive properties of FW for the progression of WMH. Elevated MD_Tissue_ and decreased FA_Tissue_, each corrected for FW, also preceded the development of new WMH. However, we did not detect changes in FA_Tissue_ over time. Of these DTI metrics, baseline FW provided the best discriminatory index for predicting the local progression of WMH penumbra into new WMH, improving upon the classification performance of clinical measures alone. As expected, since WMHs were segmented and confirmed using FLAIR images, the normalized FLAIR signal increased as new WMH developed.

Locally elevated and rising FW over time likely reflects the accumulation of interstitial fluid within NAWM tissue. The accumulation of interstitial fluid contributes to the conspicuity of WMH on FLAIR. An accumulation of FW may represent fluid shifts from the vascular space to the interstitial space, which might occur in the setting of increased vascular permeability, reduced clearance, or both. Prior *in vivo* studies have demonstrated increased BBB permeability in chronic SVD (5), and pathology studies have found excess fibrinogen deposition in chronic cerebrovascular lesions, supporting this hypothesis (34, 35). Alternatively, reduced clearance could occur in the setting of venous pathology, and prior studies have found an association between elevated FW and venous disruption on susceptibility-weighted MRI at 7 Tesla in patients with SVD (36). Pathology studies have also found more venous collagenosis associated with a higher WMH burden (7). More recently, a role for impaired glymphatic drainage has also been proposed for the pathogenesis of WMH lesions (37). Arteriolosclerosis is the most common pathologic correlate to WMH on imaging, (38) and arteriolar stiffening, or reduced compliance, impairs vasomotion and paravascular fluid clearance, thereby resulting in interstitial fluid accumulation (39). These phenomena may also be reflected by enlarged perivascular spaces (PVS), which are also associated with SVD risk factors, SVD imaging markers, and cognition in SVD and stroke patients (40–43). The FW fraction likely partially reflects fluid in the PVS since most PVS are below the resolution of diffusion imaging.

Previous studies of middle-aged to older adult community populations have also demonstrated reduced integrity of NAWM in the WMH penumbra, including reduced FA and cerebral blood flow and increased FLAIR intensity, MD, and FW (9, 12, 15, 16, 20). A cross-sectional study recently demonstrated increased white matter FW extending 8 mm from the edge of FLAIR WMH in a large cohort of community participants with elevated cardiovascular risk (20). Longitudinal studies have also shown that reduced FA and cerebral blood flow and increased normalized FLAIR intensity and MD precede the development of WMH over time (10, 15, 17). Khan et al. (44) also demonstrated DTI changes preceding new WMH development from 3 months to 1 year after ischemic stroke. By applying a 3-tissue diffusion signal composition to DTI images, they found that white matter progressing to new WMH already demonstrated more CSF-like and gray matter-like signals and less white matter-like signals at baseline compared to non-progressing NAWM (44). In this study, we build upon these findings by demonstrating that elevated FW precedes progression of NAWM into WMH and provides better discriminatory classification than tissue DTI measures, suggesting that accumulation of interstitial fluid plays a key pathogenic role in the development of new WMH lesions.

While baseline FA_Tissue_ was lower in the penumbra voxels progressing to WMH than those that did not progress, FA_Tissue_ was lower still in the NAWM and lowest in chronic WMH. This discrepancy has been demonstrated previously and is due to normal anatomical variation in FA: more central and periventricular white matter bundles (where WMH predominates) tend to be more heavily myelinated than fibers located closer to the cortical surface (9, 12, 20). Normalizing FA to an FA atlas of healthy controls can circumvent the anatomical variation in FA (16), although we did not find reports using this approach with FA_Tissue_ measures.

Normalized FLAIR intensity has also been identified as a sensitive, continuous measure for cerebrovascular disease. Rather than dichotomizing FLAIR images as lesions or NAWM, FLAIR intensity provides complementary information to DTI in predicting new lesions (16). In this study, we used normalized FLAIR intensity to reliably identify WMH growth. Most prior studies performed lesion segmentation on FLAIR and then subtracted lesion masks. To reduce the risk of false positive changes, we examined changes in normalized FLAIR intensity directly, similar to the methods developed by Schmidt et al. for the Lesion Segmentation Toolbox (32). However, rather than compare two time-point differences, we evaluated the slope over multiple time points. In this manner, we identified WMHs that are reliably progressing when compared to NAWM that is not progressing. The limitations of this approach are that it may be conservative and that shorter duration fluctuations in WMH progression and regression will be missed due to the assumption of linearity. We did not find regional WMH regression in the WMH penumbra over 1 year in this cohort, but evaluation of individual time-point differences might reveal instances of WMH regression (45, 46). This method of comparing normalized FLAIR intensities voxel-wise may be less reliable in participants undergoing more dramatic brain structural changes, such as during the first 3 months after stroke (47), or over much longer time periods when significant brain atrophy is likely. However, morphometric approaches using non-linear brain alignment could be more effective for detecting voxel-wise progression, particularly when dynamic structural changes are expected.

Prior efforts to harmonize DTI acquisition and post-processing techniques for multi-site clinical studies have focused on the conventional DTI measures FA and MD, using various region of interest approaches and histogram methods to capture a whole-brain measure of SVD that best reflects clinical and cognitive status (48). Similar attempts to harmonize FW measures are underway as part of the MARK VCID network (49). Regional approaches, on the other hand, may capture more subtle tissue changes over shorter time periods. This may lend more power to understanding pathophysiology and detecting treatment effects, but it remains to be determined how closely these regional changes relate to disability, cognitive performance, or progression to dementia.

While the strengths of this study include multiple, frequent follow-up visits over 1–2 years (median 4 visits per participant, 112 visits total), the number of participants is relatively small, which may limit generalizability and limit our analysis of clinical risk factors or other imaging markers that may also predict WMH progression. Blood pressure control, in particular, was not measured in this study but is the most significant clinical predictor for WMH progression. However, the focus of this study was to compare the predictive value of DTI metrics within at-risk white matter regions for classifying the voxel-wise progression of WMH, for which the sample size was 953,568 voxels. Finally, while we investigated WMH remote from chronic infarction as an indicator of underlying SVD, we cannot exclude subclinical cardioembolic or atheroembolic events as additional contributors to WMH progression. While prior studies found that WMH progressed faster in the setting of prior small vessel stroke, (50) our sample size was likely insufficient to identify differential progression due to index stroke etiology.

While prior studies have evaluated longitudinal changes in FA and MD in NAWM progressing to WMH, here, we also identify longitudinal changes in NAWM FW, which has recently been reported to be more closely associated with SVD disability and cognitive processing speed than other imaging markers of SVD (19). However, we estimated FW by applying a bi-tensor model to DTI with a single high B-value, which requires additional assumptions, parameter initialization, and spatial regularization (27, 28). Single-shell FW methods have also been shown to be affected by tissue perfusion that may result in an overestimation of the FW content (51). Multi-shell DTI would provide a more robust FW measure but requires longer scan times. For this reason, clinical DTI sequences are typically single-shell, so most of the recent studies using DTI FW in cerebrovascular disease have used similar approaches and yet have still demonstrated robust clinical relevance for the single-shell-derived FW measure.

## Conclusion

By evaluating voxel-wise longitudinal changes in white matter at risk for progression to WMHs in participants with chronic stroke, we identify FW as the most predictive DTI measure for progression, indicating that accumulation of interstitial fluid is an early event in the progression of white matter damage.

## Data availability statement

The datasets presented in this article are not readily available because data will be made available upon reasonable request to the corresponding author after close-out and publication of the parent study under a formal data sharing agreement and with approval from the requesting researcher's local Ethics Committee. Requests to access the datasets should be directed to rleigh4@jhu.edu.

## Ethics statement

The studies involving humans were approved by National Institutes of Health Institutional Review Board (IRB #18N0020). The studies were conducted in accordance with the local legislation and institutional requirements. The participants provided their written informed consent to participate in this study.

## Author contributions

KK: conceptualization, investigation, data curation, methodology, software, formal analysis, manuscript original draft preparation, and visualization. MZ: project administration, protocol development, and protocol supervision. RG: supervision, formal analysis review, and manuscript review and editing. CW: protocol supervision, formal analysis review, and manuscript review and editing. RL: funding acquisition, protocol development, protocol supervision, project supervision, investigation, methodology, formal analysis, and manuscript review and editing. All authors contributed to the article and approved the submitted version.
