# Supplementary material for: Diffusion tensor free water MRI predicts progression of FLAIR white matter hyperintensities after ischemic stroke
Source: Front Neurol. 2023 Sep 22;14:1172031. doi: 10.3389/fneur.2023.1172031 (PMC10559725; doi:10.3389/fneur.2023.1172031)

Supplementary Material

Diffusion Tensor Free Water MRI to Predict Progression of FLAIR White Matter Hyperintensities After Ischemic Stroke

**Kyle C. Kern,^1^ MD MS, Marwah Zagzoug,^1^ PhD, Rebecca F. Gottesman,^1^ MD PhD, Clinton B. Wright,^1^ MD MS, Richard Leigh,^1,2^ MD**

*** Correspondence:**Richard Leigh, MD
[rleigh4@jhu.edu](mailto:rleigh4@jhu.edu)
600 N Wolfe Street
Phipps 4, Suite 446
Baltimore, MD 21287

# Supplementary Methods

S.1.1 Detailed MRI Protocol
For each participant, brain MRIs were acquired serially on the same MRI scanner at one of two hospital sites: a 3T Skyra scanner (Siemens, Erlangen, Germany) or 3T Achieva scanner (Phillips, Best, the Netherlands). For the Skyra scanner, sequences included whole-brain echo-planar DTI acquired in 12 noncollinear directions at a b-value of 1000 s/mm^2^ and four non-directional images with a b-value of 0 s/mm^2^ [Repetition time (TR) = 7800 ms, Echo time (TE) = 81 ms, Matrix = 128 x 128, resolution 1.7 x 1.7 mm, slice thickness = 3.5 mm, Number of excitations (NEX) = 2]. Additional sequences included a 3D T1 [TR/TE/TI = 2300/2.95/900 ms, Matrix = 240 x240, resolution 1 x 1 x 1.2mm], a 3D FLAIR [TR/TE/TI = 4800/352/1550 ms, Matrix = 256 x 256, resolution 1 x 1 x 1mm], an axial FLAIR [TR/TE/TI = 9000/122/2500 ms, Matrix = 156 x 156, resolution = 0.86 x 0.86 mm, 3.5mm slice thickness], and a susceptibility-weighted image [TR/TE = 27/20 ms, Matrix = 256 x 256, resolution 0.86 x 0.86 mm, 1.5mm slice thickness]. DTI for the Achieva included 15 directions at b = 1000 s/mm^2^ and 1 b = 0 image [TR/TE = 4571/63 ms, Matrix = 118 x 118, resolution 2 x 2 mm, slice thickness = 3.5 mm, NEX = 2]. Additional scans included a 3D T1 [TR/TE = 9.86/4.59 ms, Matrix = 256 x 256, resolution 0.94 x 0.94 x 1mm], 3D FLAIR [TR/TE/TI = 8000/347.8/2400 ms, Matrix = 256 x 256, resolution 0.98 x 0.98 x 1mm], an axial FLAIR [TR/TE/TI = 9000/120/2600 ms, Matrix = 210 x 210, resolution = 1.1 x 1.1 mm, 3.5mm slice thickness], and SWI [TR/TE: 30/20 ms, Matrix 300 x 300, resolution 0.57 x 0.57 mm, 2mm slice thickness].

S.1.2 DTI Processing and Free Water Calculation
DTI data was corrected for eddy current distortions and motion using FSL’s Eddy (26) and a tensor was fit at each voxel. A single-shell free water (FW) model was applied using DIPY. (24,27,28) To generate FW maps, a two-compartment model was fit that included the tissue tensor and an isotropic FW compartment with the diffusivity of water (3x10^-3^ mm^2^/s at 37 degrees) as described by Pasternak et. al.(27) Fitting was accomplished using a spatially regularized gradient descent algorithm. Parameters were initialized using a hybrid model that incorporates the B0 signal and the conventionally calculated MD map as described by Golub et. al. (28) The resulting images included the free water fraction, fractional anisotropy corrected for FW (FA_Tissue_), mean diffusivity corrected for FW (MD_Tissue_) and a trace image calculated as the geometric mean of the diffusion weighted images.

S.1.3 Automatic Lesion and Tissue Segmentation
T1, 3D FLAIR and axial FLAIR were bias-field corrected and used to segment WMH, for each timepoint in native space using FreeSurfer-based Sequence Adaptive Multimodal SEGmentation (SAMSEG). (22) SAMSEG uses multiple image contrasts and a generative model to provide an automated, whole-brain neuroanatomical segmentation including WMH lesions. SAMSEG was also used to create white matter, gray matter, and CSF masks for the average T1 for each participant. Segmentations were visually inspected for accuracy. The lesions at any timepoint were combined across time, the summation mask was dilated by 3 mm, and then the baseline WMH were subtracted to create a WMH penumbra of both baseline and de novo lesions. The remaining non-lesion, non-penumbra white matter comprised the normal appearing white matter (NAWM) mask that remained stable throughout the study period.

# S.1.4 Sensitivity Analysis The primary analysis used DTI measurements that were extracted after alignment to a midpoint T1 space. However, since interpolation can affect DTI quantities, we repeated the analysis after extracting DTI metrics from the native, unaligned images. To accomplish this, we transformed the white matter ROIs back into native DTI space. White matter regions included normal appearing white matter (NAWM) that remained stable throughout the study period, WMH penumbra that remained stable, WMH penumbra that progressed to new WMH, and baseline WMH lesions. A linear inverse transformation was calculated from the midpoint T1 space for each subject back to the T1 space for each timepoint. A nonlinear inverse transformation was calculated from the native T1 space for each timepoint back to the native DTI space for each timepoint. The two inverse transformations were combined and applied simultaneously so that nearest neighbor interpolation was only applied once. With the ROI masks in native DTI space, mean DTI metrics (FW, FA_Tissue_, and MD_Tissue_) were calculated within each of the four regions for each subject and timepoint. Repeated measures ANOVA was used to determine baseline differences across regions. Mixed effects linear regression was used to test for change in each DTI metric over time across regions. Finally, each WMH penumbra voxel was classified as stable or WMH progression, and the DTI metrics at each voxel were used in a receiver-operator characteristic (ROC) curve analysis.

# Supplemental Results

S.2.1 Sensitivity Analysis: Region of Interest Approach

When derived from native DTI space, FW differed at baseline for each of the four regions (F(3, 25) = 607.6; p < 0.0001; Supplemental Figure 1). FW increased over time within the penumbra region where new lesion growth was detected (Supplemental Figure 2). FA_Tissue_ (corrected for FW) differed at baseline between all regions (F(3,25) = 88.5; p < 0.0001; Supplemental Figure 1) but did not change over time (Supplemental Figure 2). MD_Tissue_ (corrected for FW) differed at baseline between all regions [F(3, 25) = 225.5; p < 0.0001; Supplemental Figure 1]. MD_Tissue_ increased in the new lesion growth region and in the baseline WMH lesions (Supplemental Figure 2).

S.2.2 Sensitivity Analysis: ROC Curve Approach

Using logistic regression across all participants’ pooled WMH penumbra voxels, and covarying for age, sex, race, MRI site, and baseline WMH lesion volume, each DTI metric at baseline, derived in native DTI space, was associated with voxel-wise progression to new WMH lesion. Baseline voxel-wise FW provided the highest classification performance in the ROC curve analysis with an area under the curve (AUC) of 0.725 [95% confidence interval: 0.722 to 0.728], followed by MD_Tissue_ (AUC = 0.679 [0.676 to 0.682]), and then FA_Tissue_ (AUC = 0.594 [0.590 to 0.598]). Each voxel-wise DTI metric significantly improved upon the model using only clinical predictors (Supplemental Figure 3).

1. **Supplemental Figures**

Supplemental Figure 1
Sensitivity Analysis: Baseline DTI metrics derived in native DTI space differ across white matter regions, including normal appearing white matter (NAWM), white matter hyperintensity (WMH) penumbra that remained stable throughout the study (Penumbra: No Growth), WMH penumbra that progressed into new WMH lesions (WMH progression), and baseline WMH lesions. A) Baseline Free Water (FW) fraction differed across each region (p < 0.001). B) Baseline Tissue Fractional Anisotropy corrected for FW (FA_Tissue_) differed across each region (p < 0.001). C) Baseline Tissue Mean Diffusivity corrected for FW (MD_Tissue_) differed across each region (p < 0.001).


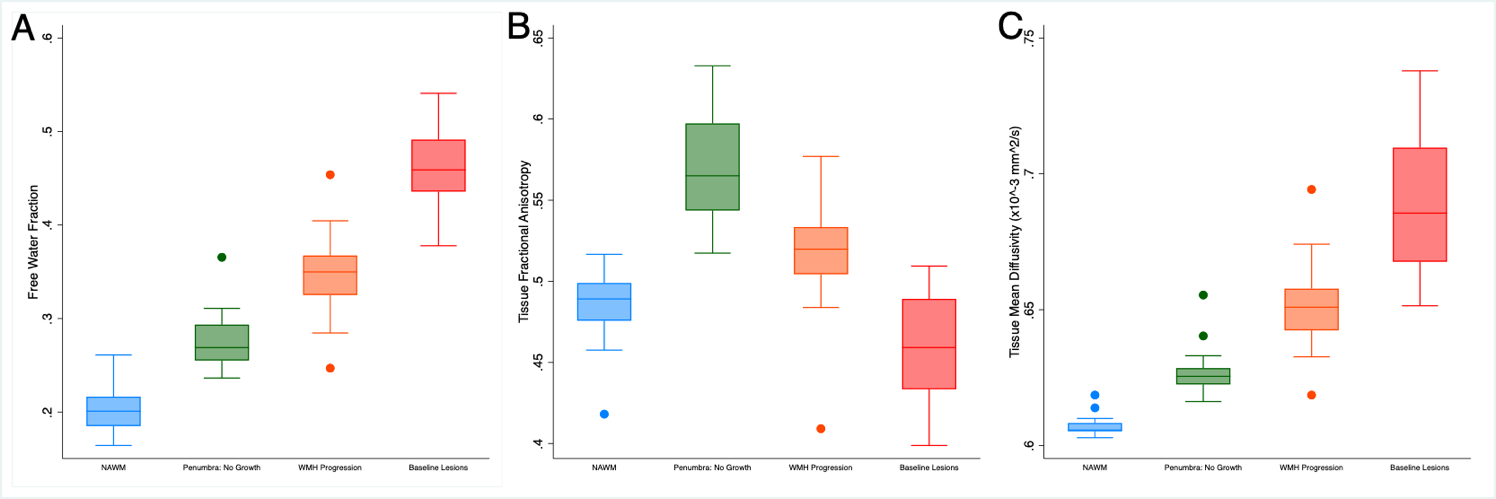


Supplemental Figure 2
Sensitivity Analysis: Longitudinal changes in DTI metrics derived in native DTI space within each tissue region. A) Free Water (FW) fraction increased in the WMH penumbra region that progressed to new WMH lesions (WMH progression; p < 0.001). B) Tissue Fractional Anisotropy (FA_Tissue_) did not significantly change in any white matter region over time. C) Tissue Mean Diffusivity (MD_Tissue_) increased in the WMH progression region, as well as in the baseline WMH lesions (p < 0.001).


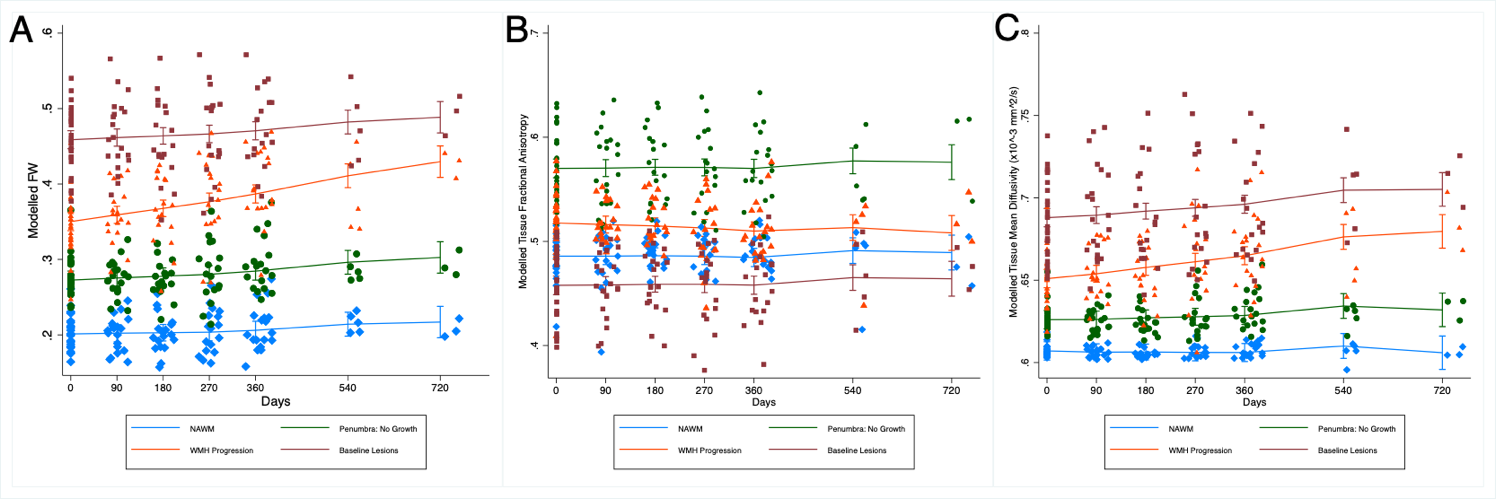


Supplemental Figure 3

Sensitivity Analysis: ROC Curve Analysis using DTI metrics (derived from native DTI space) to predict voxel-wise progression to white matter hyperintensities (WMH). White matter voxels in the WMH penumbra were classified as stable or progressing to new lesions, and the discriminatory performance of voxel-wise DTI metrics were tested using receiver-operator characteristic (ROC) curve analysis. Free Water (FW) fraction provided the best discriminatory performance in classifying WMH penumbra voxels that would progress to new WMH. FW had superior classification performance compared to Tissue Mean Diffusivity (MD_Tissue_) corrected for FW, Tissue Fractional Anisotropy (FA_Tissue_) corrected for FW, or clinical predictors alone.
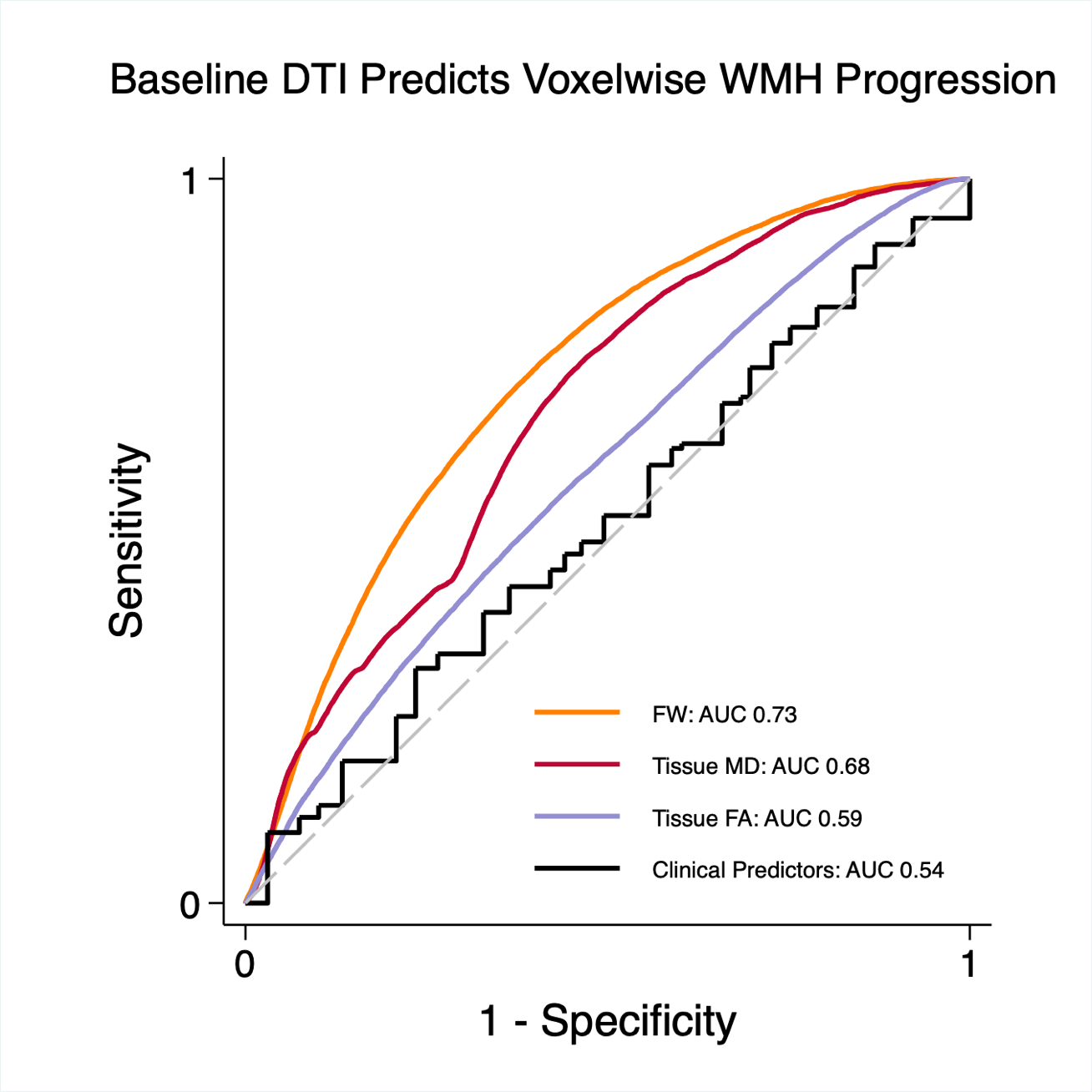

Supplement: Supplementary file 1 [file Data_Sheet_1.docx]
